# SARS-CoV-2 molecular diagnosis at airports to minimize travel-related COVID-19 spread

**DOI:** 10.1038/s41598-022-14586-z

**Published:** 2022-07-11

**Authors:** Marc-Antoine de La Vega, Ara XIII, Marc F. Lee, Gary P. Kobinger

**Affiliations:** 1 Department of Microbiology and Immunology, University of Texas Medical Branch, 301 University Blvd, Galveston, TX 77555 USA; 2 Galveston National Laboratory, Galveston, TX USA; 3 CIUSSS de La Capitale-Nationale, Québec, QC Canada

**Keywords:** SARS-CoV-2, Diagnosis, Epidemiology

## Abstract

Following the identification of SARS-CoV-2, screening for air travel helped mitigate spread, yet lessons learned from a case study of air travel within Canada display enhanced techniques to better identify infected individuals, informing future responsive screening. While international travel bans limit infectious spread beyond a country’s borders, such measures are hardly sustainable economically and infrequently address domestic travel. Here, we describe a case study from Canada, where a diagnostic laboratory at point of travel conducted real-time PCR-based detection of SARS-CoV-2 in support of existing interventions, including clinical and epidemiological questionnaires, and temperature checks. All mining workers departing from a populated urban area flying to one of two sites (Site A and B) in a remote northern Canadian region, which we deemed “at-risk”, because healthcare services are limited and vulnerable to epidemics. Data collected between June and November 2020 on 15,873 clinical samples, indicate that molecular diagnosis allowed for identification of 13 infected individuals, who would have otherwise been missed by using solely nonpharmaceutical interventions. Overall, no outbreaks, COVID-19-related or other, were detected at the point of travel up to December 2021 since the implementation of the laboratory, suggesting this screening process is an effective means to protect at-risk communities. The success of this study suggests a process more practical than travel bans or an unfocused screening of air travelers everywhere.

## Introduction

Countries had adopted multiple approaches to minimize the spread of SARS-CoV-2 from infected travelers^1,2^. For example, spread was prevented when travelers were subjected to a 14-day quarantine of susceptible individuals and isolation of SARS-CoV-2- positive individuals upon destination arrival along with follow-up by public health agents. Even so, such isolations with epidemiological and laboratory support are costly and cumbersome to sustain for extended periods, ultimately discouraging business and leisure travel. In contrast, a questionnaire that evaluates risks of infection is comparably inexpensive and logistically feasible. Still, questionnaires alone rely on the precision of memory, honesty, and a semantic understanding of each question to provide useful data, while temperature checks and other clinical assessments allow asymptomatic carriers to slip through screening^3,4^. Lastly, a reliance on epidemiological data to evaluate probabilities of infection and inform travel procedures overlook small groups, even single outliers are sufficient for transmitting pathogens and inflaming an outbreak.

Though the pandemic caused various aspects of society to shut down, critical industries such as the Canadian mining industry remained operational and required travel of essential personnel, barring an unfeasible complete restructuring of on-site living. Many mines continued to operate, including those in remote areas of northern Canada. While Canadian territories established a mandatory 2-week quarantine before traveling North, miners from the current studies were exempt since a no-contact policy with local communities was in place, including along travel routes. These northern regions contain small and scattered populations for which access to healthcare is limited and medical evacuation can account for up to 20% of their health budget^5^. As such, we recognized this group was at-risk for being vulnerable upon importation of virus, and we established a mobile diagnostic laboratory to conduct RT-PCR at the premises of the Southern Canada airport of departure (AD), an area isolated for travel to mining Sites A and B in Northern Canada. Here, we report the results from an observational study where molecular diagnostics was used successfully in the context of air travel, and allowed the identification of COVID-19 cases that otherwise would have been missed using other traditional nonpharmaceutical interventions such as questionnaires and temperature checks alone.

## Methods

### Ethics

Ethical approval for this project was sought at the *Comité central d’éthique de la recherche du ministère de la Santé et des Services Sociaux*, the Ethical Review Board for the provincial government of Quebec (Canada), which follows the Tri-Council Policy Statement for Ethical Conduct for Research Involving Humans (TCPS2, 2018). According to article 2.4 of this statement, “REB review is not required for research that relies exclusively on secondary use of anonymous information, or anonymous human biological materials, so long as the process of data linkage or recording or dissemination of results does not generate identifiable information”. Therefore, given that the data we used came from a primary outcome of human diagnostics, it was established by the *Comité central d’éthique de la recherche du ministère de la Santé et des Services Sociaux* that an REB review was not required for this publication. Therefore, ethics approval was waived by the committee. Of note, written informed consent was obtained from all participants who were sampled during the duration of the study, and all methods were performed in accordance with the relevant guidelines and regulations.

### Study population

All samples collected throughout this study were sampled on workers from the mining industry only, and due to recurring work shifts over the study period, many individuals were tested multiple times. All individuals included in this study were over 18 years old and the study population was predominantly male, although true numbers regarding the male:female ratio of tested individuals are unknown, as this information was not collected by the laboratory. However, for the year of 2020, the mining company reported a workforce at site A and B of ⁓1200 and ⁓1800 employees, respectively with a 13% female-employment rate.

### Diagnostic workflow

At the time the study was originally conducted, individuals travelling by plane to Site A and Site B were sampled at the AD, which they would reach individually by car. Once all passengers had been sampled in a dedicated area of the AD, they boarded their respective plane. Airport personnel, though limited in interaction with the miners, followed pandemic safety protocol, including their own health checks. Planes departed, one for Site A and one for Site B, and the analysis of their samples was initiated in a laboratory located directly in front of the airport, inside a mobile trailer specially designed for biocontainment. After landing at their respective airport of arrival, travelers deplaned onto the tarmac and boarded a bus to their respective sites. Given that the combined duration of the flight and the bus ride was under 4 h, which equaled the minimum time required to process the samples, workers were placed in quarantine in their individual bedrooms upon arrival to their respective sites while the analysis was being completed. Later testing workflow, after our Case Study, the mining company kept travelers socially distanced and waited until the results were known before the planes departed for Sites A and B.

### Efficacy monitoring

To monitor the efficacy of the AD laboratory, a second on-site laboratory was established in Northern Canada within the main mining camp of Site A, where all symptomatic individuals and randomly collected samples could be analyzed. Symptomatic individuals had to report themselves to the on-site clinic, where a thorough medical assessment was performed by qualified healthcare providers, along with collection of a nasopharyngeal swab for immediate analysis. The symptomatic individual was then placed in isolation until the result of the test was known. Twenty-five individuals were preselected from every flight manifest to Sites A and B for SARS-CoV-2 testing 5 days post arrival. The selection tested a proportion of passengers through a system combining the use of a random-number generator, employee request, and the switching of overly-selected individuals for new selections to ensure for a wider distribution, regardless of the number of passengers on each flight. Due to the remoteness of Site B, random sampling was not consistently performed, and efficacy monitoring relied on clinical manifestations only, where samples of symptomatic individuals were shipped to the laboratory of Site A.

### RT-PCR testing

SARS-CoV-2 genomic material was detected by a laboratory-developed test that used the Sarbeco E gene FAM label Coronavirus detection kit (TIB-MOLBIOL), the TaqMan Fast Virus 1-Step MasterMix (ThermoFisher), and either the LightCycler 96 instrument (Roche; Airport site) or the Magnetic Induction Cycler PCR Machine (Bio Molecular Systems; Site A). Briefly, nasopharyngeal swabs were collected from workers and stored in Dulbecco's modified Eagle’s medium (500 µL) containing penicillin-streptomycin (1%). Samples were extracted inside a biosafety cabinet or a closed isolator (glovebox) within three hours following collection using the QIAamp Viral RNA Mini kit (Qiagen) and processed for RT-PCR amplification according to the manufacturer’s instructions. The limit of detection of the assay was established at 2.9 copies/reaction, with a clinical sensitivity (based on 28 positives) and sensibility (based on 100 negatives) of 100%, similar to results reported by the Foundation for Innovative New Diagnostics^6^.

## Results

**Figure 1:**
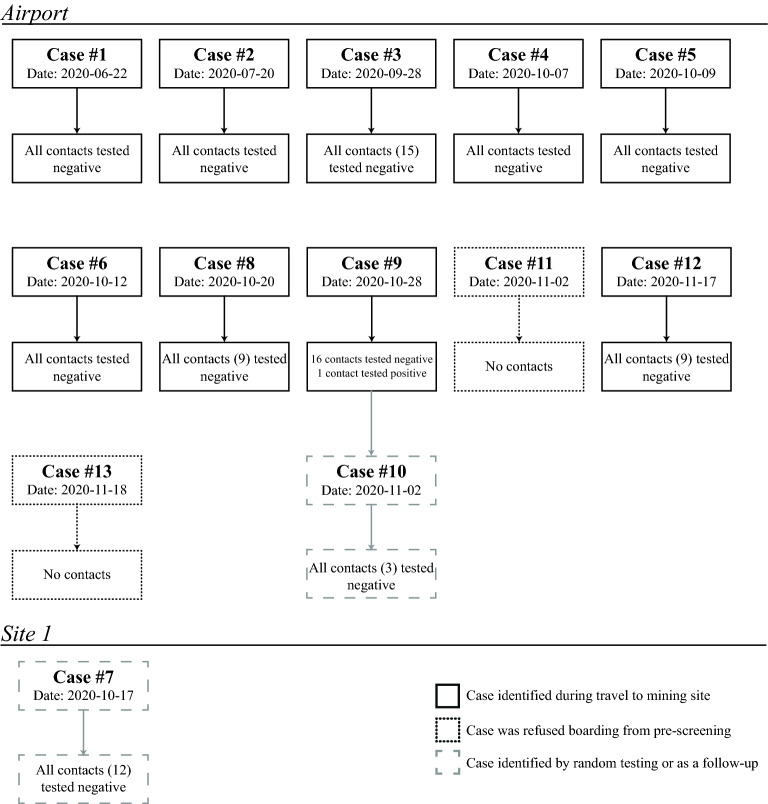
Individuals were infected with SARS-CoV-2, as identified by RT-PCR at the airport or Site A laboratory. In total, 13 individuals were identified as positive for SARS-CoV-2. Of those, 11 were identified at the airport’s laboratory, while 2 were identified at the laboratory established at Site A. Of the 11 travelers identified, one was intended for an international business trip (Case #1), two were traveling to Site B (Case #2 and #3), while the other eight individuals were either symptomatic and refused boarding, or they were scheduled to head to Site A (Case #4, #5, #6, #8, #9, #11, #12, and #13). For the remaining two cases, one was identified through random testing at Site A (Case #7), and one was identified as a close contact of Case #9 (Case #10), who sat beside each other on the plane to Site A. Interestingly, Case #1 was the very first presumptive positive case identified at the airport’s laboratory on the very first day of operations.

The airport laboratory operationally started June 22, 2020. As of November 18, 2020, the airport laboratory analyzed 15,873 samples from all travelers heading to Site A or B, representing approximately 189 flights, while the laboratory located at site A for efficacy monitoring analyzed 5765 samples. Of the 15,873 samples tested at the AD, 12 individuals were identified as presumptive positive cases, while 1 was identified at site A; all cases were confirmed by a second reference laboratory. This represents a positivity rate of 0·08% at the AD, while the Canadian rate on November 18, 2020 was 2·96%^7^. Of the 13 individuals identified as positive for SARS-CoV-2: one case tested positive before traveling internationally for business (Case #1); two individuals were traveling to Site B (Cases #2 and #3), while the remaining individuals were either refused boarding because they had declared symptoms (Cases #11 and #13) or were traveling to Site A (Cases #4, #5, #6, #8, #9, and #12); Case #10 was identified 5 days postevacuation from Site A as a follow-up to an epidemiological investigation after identification as a close contact of Case #9; and the remaining case was identified by the laboratory established at Site A, following random testing (Case #7) (Fig. 1).

## Discussion

Our study results support a two-pronged approach toward greatly reducing passage of pathogens without the impracticality of a travel ban or population-wide screening. We propose: (1.) identification of at-risk communities, defined by, (a.) environmental isolation, which accounts for enormous medical evacuation budget, (b.) work necessity, which accounts for increased interactions and transmission opportunities both intra-environmental (on-site) and from workforce influx (travelers), and (c.) limitations of healthcare, due to economic and geographic feasibility, resulting in distancing from robust healthcare access; and (2.) multifactorial preventions that include (a.) questionnaires probing exposure potential and symptoms (one prescreening travel to the AD 48 h prior, and one at AD prior to social distancing, awaiting RT-PCR results), (b.) basic prevention precautions, such as the wearing of masks and hand washing, (c.) temperature checks (at the AD and other areas of congestion, such as on-site cafeteria), (d.) RT-PCR screening through AD laboratories, and (e.) social distancing, isolation, and quarantine protocol.

Separately, the various facets of prevention have individual efficacy. For example, according to this study, the AD questionnaire alone would have prevented two of the 13 individuals (15·4%) from boarding the plane, as Case #11 and Case #13 declared either symptoms or recent contacts with a confirmed case of COVID-19 at the time of boarding. The prescreening form to be completed by travelers 24 to 48 h prior to boarding would result in individuals with symptoms or contact with a confirmed case to be referred to the public health system (and remain outside our data from swab samples). The AD questionnaire provides additional screening coverage between the prescreening form (one to two days prior) and preboarding the same day. An added benefit of the AD questionnaire within view of preboarding swabbing seems to be that individuals pay greater attention to symptoms and potential exposure when completing this questionnaire, aware of the upcoming laboratory testing (as has been voluntarily reported by individuals).

Though the prescreening and AD questionnaires are excellent tools toward mitigating infectious spread, the example of Case #12 illustrates the necessity for the RT-PCR screening, as the worker neglected to reveal in the questionnaire having returned from an international trip the day prior to boarding for Site A. Accurate information would have instituted a 14-day quarantine or isolation upon returning to Canada, as per federal regulations at the time^8^. The lack of signs and symptoms, to include elevated temperature, show the value of RT-PCR in reducing travel of asymptomatic individuals capable of causing an outbreak. Even so, one individual, Case #7, did get through all AD screening, to include RT-PCR. This case may represent a small fraction of the asymptomatic carriers in the prodromal period when the virus is under the level of detection of laboratory tests, though a false negative cannot be ruled out. Likely because of early detection at the mining site and subsequent isolation, along with other basic health protocol, Case #7 did not induce a detectable spread of SARS-CoV-2 by the healthcare workers and laboratory on Site A. Future studies to assess the efficacy of transmission prevention should consider the addition of serological surveys where economically and functionally permissible.

Our study revealed a lower breach of screening by prodromal asymptomatic carriers (1 of 13) than the 44% projected by other models, translating to an expected 5 to 6 individuals^9^. Even so, several factors should be considered mitigating comparisons: (1.) Our study is limited by the low number of cases reported (13) of 15,873 samples tested; (2.) Mining workers were in a culture of continued screening and may have been more rigorous with health-safety measures; (3.) The prescreening questionnaire 24 to 48 h prior to travel resulted in a waiting period for individuals who acknowledged exposure risk, to include travel, thereby surpassing the typical 2.4-day prodromal period upon AD screening^9^; and (4.) Our data derived from a uniformity of process rather than an amalgamation of disparate sites and processes. Furthermore, comparisons of data unrelated to prodromal asymptomatic breach are largely erroneous, particularly to sites testing much larger groups of symptomatic individuals, such as hospitals and national reference centers.

Though no secondary transmission was detected at either mining site as of December 2021, Case #9 was associated with Case #10 due to inflight seating proximity. This potential exposure route is now mitigated by the revised protocol whereby boarding occurs after RT-PCR data is returned. Overall, the current report supports the use of diagnostic laboratories at points of travel to provide an added contribution to other control measures. Though the implementation costs of such measures were entirely assumed by the private mining company; the laboratory costs and scientific expertise of the screening process remained low, having been conducted by a not-for-profit organization. Even so companies and organizations assuming the expenses of such combined screening measures should weigh those costs against the expense of pandemic spread and the associated medical response and industry shutdown, let alone the ethical implications of employing lesser safety precautions.

## Data Availability

The datasets generated and/or analyzed during the current study are available from the corresponding author upon reasonable request.
